# Брадикинин и ангиотензин-превращающий фермент в крови больных с диабетической ретинопатией и прогноз развития диабетического макулярного отека (пилотное исследование)

**DOI:** 10.14341/probl12762

**Published:** 2021-08-19

**Authors:** В. В. Нероев, Н. Б. Чеснокова, О. А. Кост, Т. Д. Охоцимская, Т. А. Павленко, О. В. Безнос, П. В. Биневский, О. А. Лисовская

**Affiliations:** Национальный медицинский исследовательский центр глазных болезней имени Гельмгольца; Национальный медицинский исследовательский центр глазных болезней имени Гельмгольца; Московский государственный университет имени М.В.Ломоносова; Национальный медицинский исследовательский центр глазных болезней имени Гельмгольца; Национальный медицинский исследовательский центр глазных болезней имени Гельмгольца; Национальный медицинский исследовательский центр глазных болезней имени Гельмгольца; Московский государственный университет имени М.В.Ломоносова; Национальный медицинский исследовательский центр глазных болезней имени Гельмгольца

**Keywords:** диабетическая ретинопатия, макулярный отек, брадикинин, ангиотензин-превращающий фермент, калликреин-кининовая система, ренин-ангиотензиновая система

## Abstract

ОБОСНОВАНИЕ. Диабетический макулярный отек (ДМО) — осложнение диабетической ретинопатии, приводящее к снижению зрения. В патогенезе ДМО важную роль играют компоненты ренин-ангиотензиновой и калликреинкининовой систем: ангиотензин-превращающий фермент (АПФ) и брадикинин (Бк).ЦЕЛЬ. Определить концентрацию Бк, концентрацию и активность АПФ в сыворотке крови больных пролиферативной диабетической ретинопатией (ПДР) и оценить значимость их определения для ранней диагностики ДМО.МАТЕРИАЛЫ И МЕТОДЫ. В исследование включены две группы больных сахарным диабетом 2 типа с ПДР с наличием ДМО (n=9) и без него (n=27). Контрольная группа (n=14) — добровольцы сопоставимого возраста без диабета и офтальмопатологии. Уровни Бк и АПФ в сыворотке крови определяли методом иммуноферментного анализа, активность АПФ — по скорости гидролиза специфического субстрата.РЕЗУЛЬТАТЫ. У пациентов с ПДР без ДМО концентрация Бк в крови составила 12,00 (8,87; 13,99) пг/мл и не отличалась от нормы. У всех пациентов с ДМО содержание Бк превышало норму и составило 14,69 (13,68; 16,78) пг/мл (p<0,01). Концентрация и активность АПФ у больных ПДР без ДМО повышены в среднем на 36% и 45% соответственно, при наличии ДМО концентрация АПФ увеличена на 27%, а активность АПФ не отличалась от нормальной. Отношение концентрации Бк к концентрации и активности АПФ у больных ПДР без ДМО снижено относительно нормы, а при наличии ДМО — повышено.ЗАКЛЮЧЕНИЕ. В крови больных с ДМО содержание Бк повышено, а без ДМО находится на уровне нормы, тогда как активность и концентрация АПФ выше у пациентов с ПДР без ДМО. Следовательно, при ДМО синтез Бк превалирует над его распадом, что способствует развитию ДМО. Отношение концентраций Бк к концентрации и активности АПФ при ДМО значительно возрастает, а при его отсутствии — падает. Таким образом, для прогнозирования развития ДМО и выявления его на ранних стадиях можно использовать измерение содержания Бк в крови больных ПДР. Еще более надежную информацию о развитии ДМО может дать измерение содержания или активности АПФ и вычисление отношения концентрации Бк к концентрации или активности АПФ.

## Abstract (en)

BACKGROUND. Diabetic macular edema (DME) is a microvascular complication of diabetic retinopathy. One of the key roles in the pathogenesis of DME may belong to the components of rennin-angiotensin and kallikrein-kinin systems: bradykinin (Bk) and angiotensin-converting enzyme (ACE).PURPOSE. To determine the Bk and ACE concentration and ACE activity in serum of patients with proliferative diabetic retinopathy (PDR) and to estimate the significance of these parameters for the early diagnostic and prognosis of DMO.MATERIALS AND METHODS. Serum was collected from the 2 groups of patients with II type diabetes. Group I (n=9) had DME, group II (n=27) had PDR without DME. Control group (n=14) consisted of adult volonteers without diabetes and ophthalmic diseases. Concentration of Bk and ACE was measured using ELISA kits, ACE activity was determined enzymatically with specific fluorogenic substrate.RESULTS. Concentration of Bk in serum of patients without DME did not differ from one in controls (12,00 (9,70; 12,40) pg/ml) while all patients with DME had Bk level of 14,69 (13,68; 16,78) pg/ml that was significantly higher (p<0,01). In patients without DME ACE concentration (88,60 (77,30; 97,45) ng/ml) and ACE activity (6,8 (5,1;7,1) nmol/min·ml) were higher than normal (p<0,01) while in the case of DME concentration of ACE increased (77,36 (70,24; 86,29 ng/ml, p<0,01) and activity remained normal. The Bk/ACE concentrations ratio decreased in patients without DME and increased in those having DME.CONCLUSION. Patients with DME have increased Bk concentration along with nearly normal ACE concentration that indicate predominance of Bk synthesis over its degradation that may lead to the DME development. The Bk/ACE ratio decrease in patients with uncomplicated PDR and increase significantly in ones with DME. It means that determination of Bk in serum of patients with PDR may be used for the prediction of DME development. The Bk/ACE concentrations ratio may be even more informative.

## ОБОСНОВАНИЕ

Диабетическая ретинопатия является микрососудистым осложнением сахарного диабета, развивается последовательно от изменений, связанных с повышенной проницаемостью и окклюзией ретинальных сосудов, до появления новообразованных сосудов и фиброглиальной ткани, переходя в стадию пролиферативной диабетической ретинопатии (ПДР). Диабетический макулярный отек (ДМО) — осложнение диабетической ретинопатии, выражающееся утолщением сетчатки, накоплением жидкости в межклеточном пространстве нейроэпителия вследствие нарушения проницаемости внутреннего гематоретинального барьера и несоответствия между выходом жидкости из кровотока и способностью клеточных структур сетчатки к ее реабсорбции. Согласно результатам популяционных исследований, его распространенность варьирует от 0 до 3% при первичной постановке диагноза и возрастает до 28% при стаже заболевания более 25 лет [1]. При этом общая заболеваемость диабетической ретинопатией в России в динамике 2013–2016 гг. составила 3830,9–3805,6 и 1586,0–1497,0 на 10 000 взрослых больных при сахарном диабете 1 и 2 типа соответственно [2].

Результаты исследования Wisconsin Epidemiological Study of Diabetic Retinopathy (WESDR, 2009) с длительным периодом наблюдения показали, что при продолжительности заболевания сахарным диабетом I типа более 25 лет ДМО развивается у 29% пациентов, а клинически значимая форма — в 17% случаев. При этом через 10 лет от начала заболевания ДМО возникает у 20,1% пациентов в возрасте до 30 лет на момент выявления сахарного диабета (раннее начало) и у 39,3% пациентов с дебютом заболевания после 30 лет [3]. В большинстве развитых стран ДМО является одной из ведущих причин снижения зрения у взрослых людей трудоспособного возраста [4]. Учитывая рост заболеваемости диабетом во всем мире, проблема прогноза, ранней диагностики и лечения ДМО является социально значимой.

В патогенезе диабетической ретинопатии и в развитии ДМО большую роль играют протеолитические системы — ренин-ангиотензиновая (РАС) и калликреин-кининовая (ККС), которые находятся в тесном взаимодействии. При диабете в тканях глаза активируются обе системы [5][6]. Обе они влияют на содержание в тканях глаза VEGF, который рассматривается как один из ведущих факторов развития ДМО, а анти-VEGF-терапия в настоящее время является первой линией лечения ДМО [7].

Активация РАС выражается в увеличении активности ангиотензин-превращающего фермента (АПФ), который преобразует малоактивный пептид ангиотензин-I в ангиотензин-II. Помимо вазоконстрикторного действия, ангиотензин-II обладает провоспалительными и стимулирующими выработку ангиогенного фактора VEGF свойствами [8]. Блокада РАС способствует снижению содержания VEGF и усилению эффективности анти-VEGF-терапии. Активация ККС за счет увеличения активности калликреина плазменного типа приводит к повышению синтеза брадикинина (Бк). Бк расширяет сосуды за счет увеличения образования монооксида азота NO и простагландинов, увеличивает их проницаемость, создавая угрозу образования отеков [5][9].

Проницаемость сосудистой стенки ККС может регулироваться благодаря влиянию и на VEGF, и на Бк [10][11]. Известно, что не у всех пациентов с ДМО блокада VEGF оказывается эффективной. В качестве альтернативы для таких пациентов в настоящее время рассматриваются ингибиторы плазменного калликреина [12].

Содержание Бк определяется скоростью как его образования под действием калликреинов, так и его распада, которая во многом зависит от АПФ, так как этот фермент расщепляет Бк до неактивных пептидов. Кроме того, взаимодействие РАС и ККС осуществляется на уровне рецепторов. Так, ангиотензин-2 увеличивает экспрессию рецепторов В1 к Бк, опосредующих его провоспалительное действие [13][14].

Нами ранее было показано, что активность АПФ в крови больных диабетической ретинопатией увеличена, и степень увеличения коррелирует со стадией заболевания [15]. Однако при исследовании концентрации АПФ в крови у больных ПДР с ДМО мы не выявили повышения содержания этого фермента [16]. Поэтому возник вопрос о том, как изменяется активность АПФ в крови у больных с ДМО. Нами было сделано предположение, что отсутствие повышения концентрации АПФ в крови у больных диабетической ретинопатией может способствовать увеличению концентрации Бк вследствие уменьшения его распада.

## ЦЕЛЬ

Определить в крови больных ПДР при наличии ДМО в сравнении с больными ПДР без ДМО содержание Бк, активность и содержание АПФ, а также соотношение этих показателей для выяснения возможности прогнозирования развития ДМО у больных ПДР.

## МЕТОДЫ

Место и время проведения исследования

Исследование проведено на базе ФГБУ «НМИЦ глазных болезней им. Гельмгольца» Минздрава России и ФГБОУ ВО «МГУ имени М.В. Ломоносова» (химический факультет, кафедра химической энзимологии).

Дизайн исследования

В пилотное проспективное наблюдательное моноцентровое контролируемое нерандомизированное исследование включены пациенты с ПДР, проходящие лечение в НМИЦ глазных болезней им. Гельмгольца.

Критерии соответствия

Возраст пациентов старше 18 лет. Сахарный диабет 2 типа. Билатеральный или монолатеральный ДМО (толщина сетчатки в центральной зоне ≥350 мкм). Отсутствие клинических признаков прогрессирования ПДР (отсутствие преретинальных и интравитреальных геморрагий и прогрессирования неоваскуляризации). Всем пациентам более чем за 1 год до включения в исследование была проведена панретинальная лазеркоагуляция, у всех достигнута стабилизация пролиферативного процесса.

Критерии исключения

Другие заболевания глаз, проведенная ранее антиангиогенная терапия, декомпенсация сахарного диабета (HbA1с≥10%) или гипертонической болезни, прием ингибиторов АПФ и антагонистов рецепторов ангиотензина II (AT1-подтип).

Продолжительность исследования

Срок проведения исследования: июнь 2019 г. — февраль 2020 г.

Описание медицинского вмешательства

Пациентам проводили комплексное клинико-функциональное обследование, включающее, помимо стандартного офтальмологического обследования, оптическую когерентную томографию. У всех пациентов отбирали пробы крови из локтевой вены по стандартной методике для биохимического анализа крови в процессе лечения. Сыворотка той же крови использовалась для определения активности АПФ, концентрации АПФ и Бк.

Основной исход исследования

Оценивались концентрации АПФ и Бк и активность АПФ в сыворотке крови в обследуемых группах.

Анализ в подгруппах

Пациенты был разделены на 2 группы. У всех пациентов диагностирован сахарный диабет 2 типа, средняя длительность заболевания на момент осмотра — 11,8 года (8,9–13,9 лет), HbA1с — 7,3% (6,1–8,4).

Первую группу составили 27 пациентов с ПДР без ДМО (8 мужчин, 19 женщин; возраст от 32 до 75 лет). Вторую группу составили 9 пациентов с ПДР и ДМО (2 мужчин, 7 женщин; возраст от 45 до 71 года).

Контрольной группой послужили 14 человек без сахарного диабета и офтальмопатологии, сопоставимые по возрасту с группами больных.

Методы регистрации исходов

В сыворотке крови определяли концентрацию Бк и АПФ методом иммуноферментного анализа с помощью диагностических наборов ELISA kit for bradykinin (human) и ELISA kit for ACE (human) (Cloud-Clone Corp, США). Оптическую плотность образцов определяли с помощью многофункционального фотометра для микропланшет Synergy MX (Bio Tek, США).

Активность АПФ в сыворотке крови оценивали по начальным скоростям гидролиза субстрата N-карбобензокси-L-фенилаланил-L-гистидил-L-лейцин (Cbz-Phe-His-Leu, Serva, Германия) флуориметрическим методом [17] с регистрацией на многофункциональном фотометре для микропланшет Infinite M-200 (Tecan, Австрия).

После центрифугирования в КДЛ НМИЦ глазных болезней им. Гельмгольца аликвоты сыворотки крови для определения концентраций АПФ и Бк хранились при -20°С до проведения анализа. Определение концентрации АПФ и Бк проводили в НМИЦ ГБ им. Гельмгольца. Для измерения активности АПФ охлажденные до +5–8°С аликвоты сыворотки доставлялись в МГУ им. М.В. Ломоносова в термоконтейнере в день забора крови.

Этическая экспертиза

В связи с тем, что пациентам, включенным в исследование, не проводилось никаких манипуляций, не входящих в стандарт обследования, Этический комитет постановил, что проведения этической экспертизы не требуется (выписка 54/7 из протокола заседания Этического комитета ФГБУ «НМИЦ глазных болезней им. Гельмгольца» Минздрава России №54 от 13.05.2019).

Статистический анализ

Статистический анализ проводили при помощи пакета прикладных программ Microsoft Office Excel, входящих в стандартный комплект Microsoft Office, а также статистического пакета STATISTICA 10.0 (Stat Soft Inc., США). Применялись общепринятые методы статистической обработки: количественные показатели с ненормальным распределением представлены в виде медианы и 25 и 75 квартилей (Me (Q25; Q75)). Ввиду небольшого объема выборок при сравнении групп использовали непараметрический U-критерий Манна–Уитни. Различия считали достоверными при p<0,05.

## РЕЗУЛЬТАТЫ

На рисунках 1 и 2 приведена типичная картина состояния сетчатки при ПДР без ДМО и при наличии ДМО по данным оптической когерентной томографии.

**Figure:**
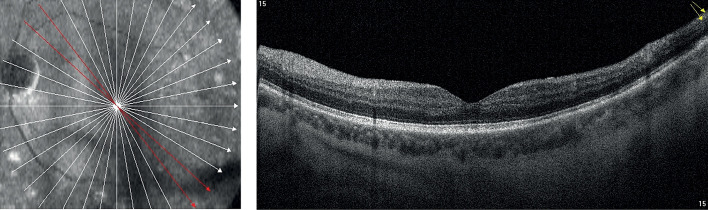
Рисунок 1. Сетчатка при пролиферативной диабетической ретинопатии без признаков отека. нейроэпителия.

**Figure:**
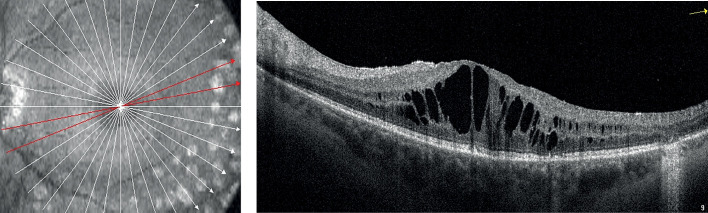
Рисунок 2. Кистовидный макулярный отек.

Проведенные нами исследования концентрации и активности АПФ и концентрации Бк в крови пациентов с ПДР свидетельствуют о том, что имеются существенные различия в содержании и активности компонентов РАС и ККС в крови больных ПДР без ДМО и при наличии ДМО (табл. 1).

Если в группе пациентов с ПДР без ДМО содержание Бк практически не отличалось от уровня контрольной группы, то в группе пациентов с ПДР при наличии ДМО содержание Бк было увеличено у всех без исключения пациентов и в среднем превышало контрольное на 25% (p=0,004).

Содержание АПФ, напротив, было в большей степени повышено у больных ПДР без ДМО (в среднем на 36%, p=0,001), а при наличии ДМО повышение оказалось меньшим, но также статистически достоверным (в среднем на 27%, p=0,043). При этом активность АПФ у больных с ДМО не отличалась от контрольной группы, тогда как у больных ПДР без ДМО повышалась на 45%.

Особенно четко прослеживается разница в соотношениях показателей. В таблице 2 приведены усредненные значения отношений концентрации Бк к концентрации и активности АПФ у тех пациентов, у которых были одновременно определены все три показателя.

Если у больных ПДР без ДМО происходит снижение отношения концентрации Бк к концентрации АПФ, то при наличии ДМО это соотношение возрастает. В группе больных ПДР с ДМО отношение содержания Бк к активности АПФ в 2 раза выше, чем в группе без ДМО и почти в 1,5 раза выше, чем в контрольной.

У большинства больных с ДМО оказалось снижено отношение активности АПФ к его концентрации, то есть в их крови была меньше доля активного фермента.

**Table:** Таблица 1. Содержание брадикинина, ангиотензин-превращающего фермента и активность ангиотензин-превращающего фермента в крови у пациентов с пролиферативной диабетической ретинопатией с диабетическим макулярным отеком и без него

Группа	Концентрация Бк(пг/мл)	Концентрация АПФ(нг/мл)	Активность АПФ (нмоль/мин·мл)
Контрольная группа (n=14)	12,00 (9,70; 12,40)	63,76 (57,75; 72,94)	4,7 (3,8;6,0)
ПДР без ДМО (n=27)	12,00 (8,87; 13,99)	88,60 (77,30; 97,45)*	6,8 (5,1;7,1)*
ПДР с ДМО (n=9)	14,69 (13,68; 16,78)*#	77,36 (70,24; 86,29)*	4,7 (4,4; 5,9)#

**Table:** Таблица 2. Соотношение концентрации брадикинина и концентрации ангиотензин-превращающего фермента (кАПФ) и активности ангиотензин-превращающего фермента (аАПФ) в крови у пациентов с пролиферативной диабетической ретинопатией с диабетическим макулярным отеком и без него

группа	(Бк/кАПФ)·10-4	(Бк/аАПФ)·10-2	(аАПФ/кАПФ)·10-2
Контрольная группа (n=10)	1,8 (1,6; 2,0)	2,4 (2,0;3,5)	7,0 (5,6; 8,0)
ПДР без ДМО (n=10)	1,3 (1,1; 1,6)*	1,7 (1,2;2,2)*	7,1 (6,7; 7,8)
ПДР с ДМО (n=8)	2,1 (1,8; 2,6)#	3,2 (2,3;4,3)#	6,0 (5,2; 7,8)

## ОБСУЖДЕНИЕ

Резюме основного результата исследования

В крови больных ПДР с ДМО значительно повышено содержание Бк, а активность и концентрация АПФ практически не отличаются от нормы. В крови больных ПДР без ДМО, напротив, содержание Бк практически не отличается от нормы, а активность и концентрация АПФ значительно возрастают.

Соответственно, отношение концентрации Бк к концентрации АПФ при наличии ДМО значительно возрастает относительно нормы, а при его отсутствии, напротив, падает.

Обсуждение основного результата исследования

Повышение содержания АПФ в крови больных диабетом уже описано в литературе и признано фактором, приводящим к развитию диабетической ретинопатии. Данное исследование посвящено изучению компонентов РАС и ККС при таком осложнении ПДР как ДМО. Особенностью представленной работы является одновременное определение активности и концентрации АПФ параллельно с содержанием Бк.

Нами ранее было установлено повышение активности АПФ в крови больных с диабетической ретинопатией, коррелирующее со стадией заболевания [15]. В представленной работе мы наблюдали увеличение как активности, так и концентрации АПФ в крови больных ПДР без ДМО. Однако полученные результаты показали, что при развитии ДМО активность и содержание АПФ в крови больных мало изменяются. Это может способствовать отмечаемому нами значительному повышению содержания Бк. Установлено, что при диабете в крови повышается содержание плазменного калликреина [18], за счет чего может увеличиваться концентрация Бк. В то же время повышенное содержание АПФ может приводить к усиленному распаду Бк до неактивных пептидов.

Отсутствие больших изменений уровня АПФ при ДМО может явиться причиной отмечаемого в этом случае значительного возрастания концентрации Бк в крови. Известно, что при ДМО активируется местное образование Бк в тканях глаза [11], но Бк может поступать в сетчатку глаза и из кровеносного русла. О том, что снижение активности АПФ может способствовать развитию отека, свидетельствует тот факт, что применение ингибиторов АПФ редко, но может приводить к ангионевротическому отеку, возникновение которого связывают со снижением скорости распада Бк [19]. Если для других органов небольшой отек может пройти незаметно, то в сетчатке он проявляется существенным снижением зрения.

В отличие от предыдущих исследований, в данной работе мы определяли одновременно концентрацию и активность АПФ в крови больных диабетической ретинопатией. Оказалось, что при ПДР без ДМО практически не изменяется соотношение этих показателей, в то время как при ДМО у большинства больных активность АПФ снижена по отношению к его концентрации. Это может быть связано с увеличением содержания в крови эндогенных ингибиторов АПФ [20].

Концентрация основного эффектора ККС Бк регулируется как активностью ККС, так и активностью РАС. По нашим данным, у больных с ПДР без ДМО доминирует РАС, тогда как при развитии ДМО, по-видимому, РАС недостаточно активна. Поэтому возникает вопрос о применении ингибиторов АПФ, часто назначаемых больным ПДР с гипертонией, при данной ситуации. Такие препараты снижают активность АПФ, способствуя повышению содержания Бк, что может привести к развитию ДМО.

Клиническая значимость результатов

Полученные данные свидетельствуют о том, что измерение содержания Бк и АПФ в крови больных сахарным диабетом может позволить определить повышенный риск развития ДМО и скорректировать методы медикаментозного лечения больных ПДР.

Кроме того, дальнейшее изучение изменений активности РАС и ККС при диабетической ретинопатии на фоне различных методов лечения может дать ответ на вопрос о причинах недостаточной эффективности некоторых из них. Например, эффективность анти-VEGF-терапии у таких больных часто оказывается меньше ожидаемой или продолжается в течение более короткого периода.

Направление дальнейших исследований

Предполагается продолжение исследований в направлении изучения влияния приема препаратов ингибиторов АПФ и блокаторов рецепторов к ангиотензину  II на биохимические показатели РАС и ККС и развитие ДМО у пациентов с диабетической ретинопатией, а также изучение активности этих систем при других методах терапии с целью прогнозирования эффективности лечения.

## ЗАКЛЮЧЕНИЕ

Получены новые данные о достоверных различиях в активности ККС и РАС у больных ПДР при наличии ДМО и без него. На основании этих данных можно полагать, что при ДМО синтез Бк превалирует над его распадом, что приводит к увеличению его содержания и способствует развитию ДМО. Следовательно, для прогнозирования возможности развития ДМО и выявления его на ранних стадиях можно использовать измерение содержания Бк в крови больных ПДР. Еще более надежную информацию о склонности к развитию ДМО может дать вычисление отношения концентрации Бк к концентрации или активности АПФ, которое при наличии ДМО значительно возрастает.

Показана роль Бк как одного из звеньев патогенеза ДМО, что дает обоснование для всестороннего изучения проблемы, включая разработку новых методов терапии и анализ возможных причин резистентности к существующим методам терапии.
